# Transition of Transient Channel Flow with High Reynolds Number Ratios

**DOI:** 10.3390/e20050375

**Published:** 2018-05-17

**Authors:** Akshat Mathur, Mehdi Seddighi, Shuisheng He

**Affiliations:** 1 Department of Mechanical Engineering, University of Sheffield, Sheffield S1 3JD, UK; 2 Department of Maritime and Mechanical Engineering, Liverpool John Moores University, Liverpool L3 3AF, UK

**Keywords:** pipe flow boundary layer, turbulent transition, large eddy simulation, channel flow

## Abstract

Large-eddy simulations of turbulent channel flow subjected to a step-like acceleration have been performed to investigate the effect of high Reynolds number ratios on the transient behaviour of turbulence. It is shown that the response of the flow exhibits the same fundamental characteristics described in He & Seddighi (J. Fluid Mech., vol. 715, 2013, pp. 60–102 and vol. 764, 2015, pp. 395–427)—a three-stage response resembling that of the bypass transition of boundary layer flows. The features of transition are seen to become more striking as the Re-ratio increases—the elongated streaks become stronger and longer, and the initial turbulent spot sites at the onset of transition become increasingly sparse. The critical Reynolds number of transition and the transition period Reynolds number for those cases are shown to deviate from the trends of He & Seddighi (2015). The high Re-ratio cases show double peaks in the transient response of streamwise fluctuation profiles shortly after the onset of transition. Conditionally-averaged turbulent statistics based on a λ_2-criterion are used to show that the two peaks in the fluctuation profiles are due to separate contributions of the active and inactive regions of turbulence generation. The peak closer to the wall is attributed to the generation of “new” turbulence in the active region, whereas the peak farther away from the wall is attributed to the elongated streaks in the inactive region. In the low Re-ratio cases, the peaks of these two regions are close to each other during the entire transient, resulting in a single peak in the domain-averaged profile.

## 1. Introduction

Unsteady turbulent flow remains a topic of interest to researchers for many years. The transient response of turbulence to unsteady flow conditions exhibits interesting underlying physics that are not generally observed in steady turbulent flows. It has the potential to give insight into the fundamental physics of turbulence, as well as holds practical importance in engineering applications and turbulence modelling. Unsteady flows are generally classified as periodic and non-periodic flows. Turbulent periodic flows have been investigated extensively over the years, both experimentally and computationally. Examples of such studies include Tu and Ramaprian [1], Shemer et al. [2], Brereton et al. [3], Tardu et al. [4], Scotti and Piomelli [5] and He and Jackson [6]. The focus of the present paper is non-periodic turbulent flows, especially concerning accelerating (or ramp-up) flows, the work of which is reviewed below.

Maruyama et al. [7] presented one of the earliest experimental investigations on the transient response of turbulence following a step change in flow. It was reported that the generation and propagation of “new” turbulence are the dominant processes in the step-increase flow cases, whereas, the decay of “old” turbulence is the dominant process in step-decrease case. He and Jackson [8] presented a comprehensive experimental investigation of linearly accelerating and decelerating pipe flows, with initial and final Reynolds numbers ranging from 7000 to 45,200 (based on bulk velocity and pipe diameter). Consistent with the earlier studies, the authors concluded that turbulence responds first in the near-wall region and then propagates to the core of the flow. It was further reported that the streamwise velocity is the first to respond in the wall region followed by the transverse components, while all components responded approximately at the same time in the core region. Overall, turbulence was shown to produce a two-stage response—an initial slow response followed by a rapid one. The behaviour of turbulence was explained by the delays associated with turbulence production, energy redistribution and propagation processes. Experimental investigation with much higher initial and final Reynolds numbers (i.e., 31,000 and 82,000, respectively, based on bulk velocity and pipe diameter) and higher acceleration rates was presented by Greenblatt and Moss [9]. It was reported that the results were in agreement with the earlier studies. In addition, the authors reported a second peak of turbulence response in a region away from the wall (at $y^{+}\;\sim \;300$). Other notable reports on the transient response of turbulence include the experimental study of He et al. [10], and the computational investigations of Chung [11], Ariyaratne et al. [12], Seddighi et al. [13] and Jung and Chung [14].

Recent numerical studies of He and Seddighi [15,16] and Seddighi et al. [17] have proposed a new interpretation of the behaviour of transient turbulent flow. It was reported that the transient flow following a rapid increase in flow rate of turbulent flow is effectively a laminar-turbulent transition similar to bypass transition in a boundary layer. With an increase in flow rate, the flow does not progressively evolve from the initial turbulent flow to a new one, but undergoes a process with three distinct phases of pre-transition (laminar in nature), transition and fully-turbulent. These resemble the three regions of boundary layer bypass-transition, namely, the buffeted laminar flow, the intermittent flow and fully developed regions, respectively. The turbulent structures present at the start of the transient, like the “free-stream turbulence” in boundary layer flows, act as a perturbation to a time-developing laminar boundary layer. Elongated streaks of high and low streamwise velocities are formed, which remain stable in the pre-transition period. In the transition period, isolated turbulent spots are generated which eventually grow in both streamwise and spanwise directions and merge with one another occupying the entire wall surface. Seddighi et al. [17] further reported that a slow ramp-type accelerating flow also shows a transitional response despite having quantitative differences in its mean and instantaneous flow. Jung and Kim [18] conducted a more comprehensive study on the effects of changing the acceleration rate and the final/initial Reynolds number ratio by systematically varying these parameters in a direct numerical simulation (DNS) study. They noted that when the increase of the Reynolds number is small or when the acceleration is mild, transition could not be clearly identified through visualisation, which was consistent with the observation by He and Seddighi [16]. The authors went further and attempted to develop a criterion for when transition could be clearly observed.

More recently, the transition nature of a transient turbulent flow starting from a turbulent flow has been demonstrated experimentally by Mathur et al. [19] in a channel, and Sundstrom and Cervantes [20,21] in a circular pipe. The former focused on the transition physics, especially the abrupt changes in the length and time scales of turbulence as the transition occurs. Their experiments were accompanied by large eddy simulations (LES) of the experiments and an analytical solution based on the extended Stokes first problem solutions for the early stages of the flow. Sundstrom and Cervantes [20] obtained an analytical solution for the pre-transition phase of an accelerating flow and demonstrated that the velocity profile possess a self-similarity during the early stages. Sundstrom and Cervantes [21] on the other hand compared experimental results of accelerating and pulsating flows. They have found that, like accelerating flows, the accelerating phase of the pulsating flow also demonstrated distinct staged development, namely, a laminar-like development followed by rapid generation of turbulence.

The DNS study presented by He and Seddighi [16] (HS15, hereafter) covered a Reynolds number range from 2800 to 12,600 (i.e., a maximum Reynolds number ratio of 4.5). The initial turbulence intensity, $Tu_{0}$, equivalent to ‘free-stream turbulence’ of boundary layer flows was thus defined by HS15, by using peak turbulence following the commencement of the transient:

(1) $$Tu_{0}=\frac{{\left(u_{rms,0}^{\prime }\right)}_{max}}{U_{b1}}\approx 0.375\frac{U_{b0}}{U_{b1}}{\left(Re_{0}\right)}^{-0.1}$$

where ${\left(u_{rms,0}^{\prime }\right)}_{max}$ is the peak r.m.s. streamwise fluctuating velocity of the initial flow; $U_{b0}$ and $U_{b1}$ are the initial and final bulk velocities, respectively; and $Re_{0}$ is the initial Reynolds number ($Re_{0}=U_{b0}\delta /\nu$, where δ is the channel half-height and ν denotes the fluid kinematic viscosity). The “turbulence intensity” range covered by HS15 was 15.4% down to 3.8%. The purpose of the present study is to extend the range of turbulence intensity or Reynolds number ratio using large eddy simulations. The present paper increases the final flow to a Reynolds number of 45000; thereby increasing the Reynolds number ratio to ~19 and decreasing the turbulence intensity to 0.9%. The effect of high Re-ratio on the overall transition process, the transitional Reynolds number and the turbulent fluctuations is presented here. The simulations are also performed on different domain sizes to investigate the effect of domain length.

## 2. Methodology

Large-eddy simulations of unsteady turbulent channel flow are performed using an *in-house* code, developed by implementing subgrid calculations on the base DNS code, *CHAPSim* [15,22]. The resulting filtered governing equations in dimensionless form read:

(2) $$\frac{\partial {\bar{u}}_{i}}{\partial t}+\frac{\partial }{\partial x_{j}}\left({\bar{u}}_{i}{\bar{u}}_{j}\right)=-\frac{\partial \bar{P}}{\partial x_{i}}+\frac{1}{Re_{c}}\frac{\partial^{2}{\bar{u}}_{i}}{\partial x_{j}\partial x_{j}}-\frac{\partial \tau_{ij}}{\partial x_{j}}$$

(3) $$\frac{\partial {\bar{u}}_{i}}{\partial x_{i}}=0$$

where the overbar ($\bar{\;}$) denotes a spatially-filtered variable, $Re_{c}$ is Reynolds number based on characteristic velocity ($Re_{c}=U_{c}\delta /\nu )$ and $\tau_{ij}$ represents the residual (or subgrid-scale) stress:

(4) $$\tau_{ij}=\bar{u_{i}u_{j}}-{\bar{u}}_{i}{\bar{u}}_{j}$$

Here, the governing equations are non-dimensionalised using the channel half-height (δ), characteristic velocity ($U_{c}$), time scale ($\delta /U_{c}$) and pressure-scale ($\rho U_{c}^{2}$). $x_{1}$, $x_{2}$, $x_{3}$ and $u_{1}$, $u_{2}$, $u_{3}$ stand for streamwise, wall-normal and spanwise coordinates and velocities, respectively. Although the characteristic velocity ($U_{c}$) used in the simulations was the centreline velocity of the laminar Poiseuille flow at the initial flow rate, the results presented here are re-scaled using the initial bulk velocity ($U_{b0}$) as the characteristic velocity. The governing Equations (2) and (3) are spatially discretized using second-order central finite-difference scheme. An explicit third-order Runge-Kutta scheme is used for temporal discretization of the non-linear terms, and an implicit second-order Crank-Nicholson scheme for the viscous terms. In addition, the continuity equation is enforced using the fractional-step method (Kim and Moin [23]; Orlandi [24]). The Poisson equation for the pressure is solved by an efficient 2-D fast Fourier transform (FFT, Orlandi [24]). Periodic boundary conditions are applied in the streamwise and spanwise directions and a no-slip boundary condition on the top and bottom walls. The code is parallelized using the message-passing interface (MPI) for use on a distributed-memory computer cluster. Detailed information on the numerical methods and discretization schemes used in the code, and its validation can be found in Seddighi [22] and He and Seddighi [15]. The subgrid-scale stress is modelled using the Boussinesq eddy viscosity assumption:

(5) $$\tau_{ij}-\frac{1}{3}\tau_{kk}\delta_{ij}=2\nu_{sgs}{\bar{S}}_{ij}$$

where $\delta_{ij}$ is Kronecker delta, $\nu_{sgs}$ is the *subgrid-scale viscosity* and ${\bar{S}}_{ij}$ is the resolved strain rate. The subgrid-scale viscosity is modelled using the WALE model of Nicoud and Ducros [25]:

(6) $$\nu_{sgs}={\left(C_{w}\Delta \right)}^{2}\frac{{\left({\bar{S}}_{ij}^{d}{\bar{S}}_{ij}^{d}\right)}^{3/2}}{{\left({\bar{S}}_{ij}{\bar{S}}_{ij}\right)}^{5/2}+{\left({\bar{S}}_{ij}^{d}{\bar{S}}_{ij}^{d}\right)}^{5/4}}$$

where ${\bar{S}}_{ij}^{d}$ is the traceless symmetric part of the square of the filtered velocity gradient tensor, ${\bar{S}}_{ij}$ is the filtered strain rate tensor, $C_{w}$ is the model constant and Δ is the filter width which is defined as ${\left(\Delta x_{1}.\Delta x_{2}.\Delta x_{3}\right)}^{1/3}$. As the above model invariant is based on both local strain rate and rotational rate of the flow, the model is said to account for all turbulent regions and is shown to even reproduce transitional flows [25].

**Figure 1:**
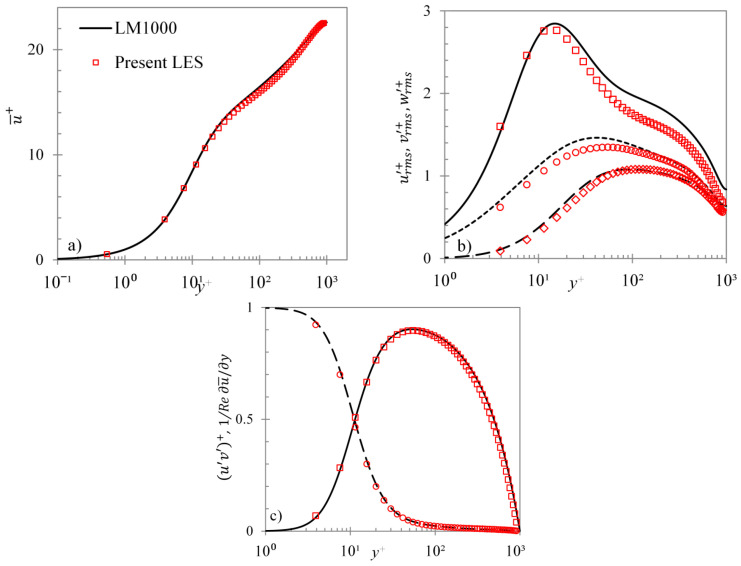
Comparison of present LES of steady channel flow at $Re_{\tau }\;\sim \;950$ with DNS of Lee & Moser (2015) $Re_{\tau }\;\sim \;1000$. (**a**) mean velocity in wall coordinates; (**b**) r.m.s. velocity fluctuations in wall coordinates (DNS: –– $u_{rms}^{\prime +}$, – – $v_{rms}^{\prime +}$, -- $w_{rms}^{\prime +}$; LES: □ $u_{rms}^{\prime +}$, ◊ $v_{rms}^{\prime +}$, ○ $w_{rms}^{\prime +}$); and (**c**) Reynolds and viscous stresses in wall coordinates (DNS: –– ${\left(u\prime v\prime \right)}^{+}$, – – 1/*Re* $\partial \bar{u}/\partial y$; LES: □ ${\left(u\prime v\prime \right)}^{+}$, ○ 1/*Re* $\partial \bar{u}/\partial y$).

**Figure 2:**
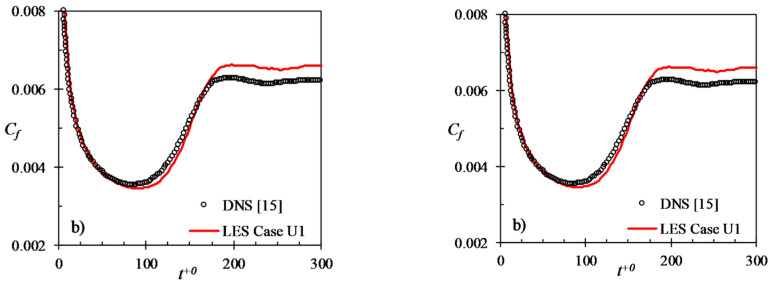
Present LES validation cases, U1 and U2, compared with the DNS cases of He & Seddighi (2013) [15].

For validation purpose, the results of the present code have been compared with DNS results. In Figure 1, steady turbulent channel flow statistics for the present code at $Re_{\tau }\;\sim \;950$ have been compared with those of Lee and Moser [26] at $Re_{\tau }\;\sim \;1000$ ($Re_{\tau }=u_{\tau }\delta /\nu$, is the frictional Reynolds number defined using the friction velocity, $u_{\tau }$, and channel half-height). It can be seen that the LES profiles are in agreement with those of DNS. It should be noted that the peak streamwise turbulent fluctuation is predicted fairly accurately by the LES, even though the predictions are less accurate away from the wall-region. A further validation of the present LES code for unsteady flow is presented in Figure 2, where two DNS accelerating flow cases of He and Seddighi [15,16] are reproduced. It is clear from the figure that the transient response of friction factor predicted by LES follows very closely that of DNS. Although the final steady value of LES is slightly higher than that of DNS (i.e., turbulence shear is slightly over-predicted), the timing of the minimum friction factor and the recovery periods are accurately predicted by the LES.

## 3. Results and Discussion

Simulations are performed for a spatially fully developed turbulent channel flow subjected to a step-like linear acceleration using large eddy simulations. Two cases (U1 and U2), as described above, have been used to validate the LES spatial resolution with that of the DNS results of He and Seddighi [15,16]. Further four cases have been designed with Reynolds number ratios up to 19. The present cases have been described in Table 1. The spatial resolution provided in the table is in wall units of the final flow. Multiple realizations have been performed for each case, each starting from a different initial flow field. The spatial resolution of the cases U3–U5 resembles that of the LES validation cases, U1 and U2. However, due to limited computational resources, the resolution of the case U6 has been restricted to lower values. It is expected that the basic physical phenomena and trend of ‘transition’ has been captured despite the lower spatial resolution. Cases U3–U6 have also been repeated with different domain lengths to ensure that there is a minimal effect of the domain length on the physical process.

### 3.1. Instantaneous Flow Features

**Figure 3:**
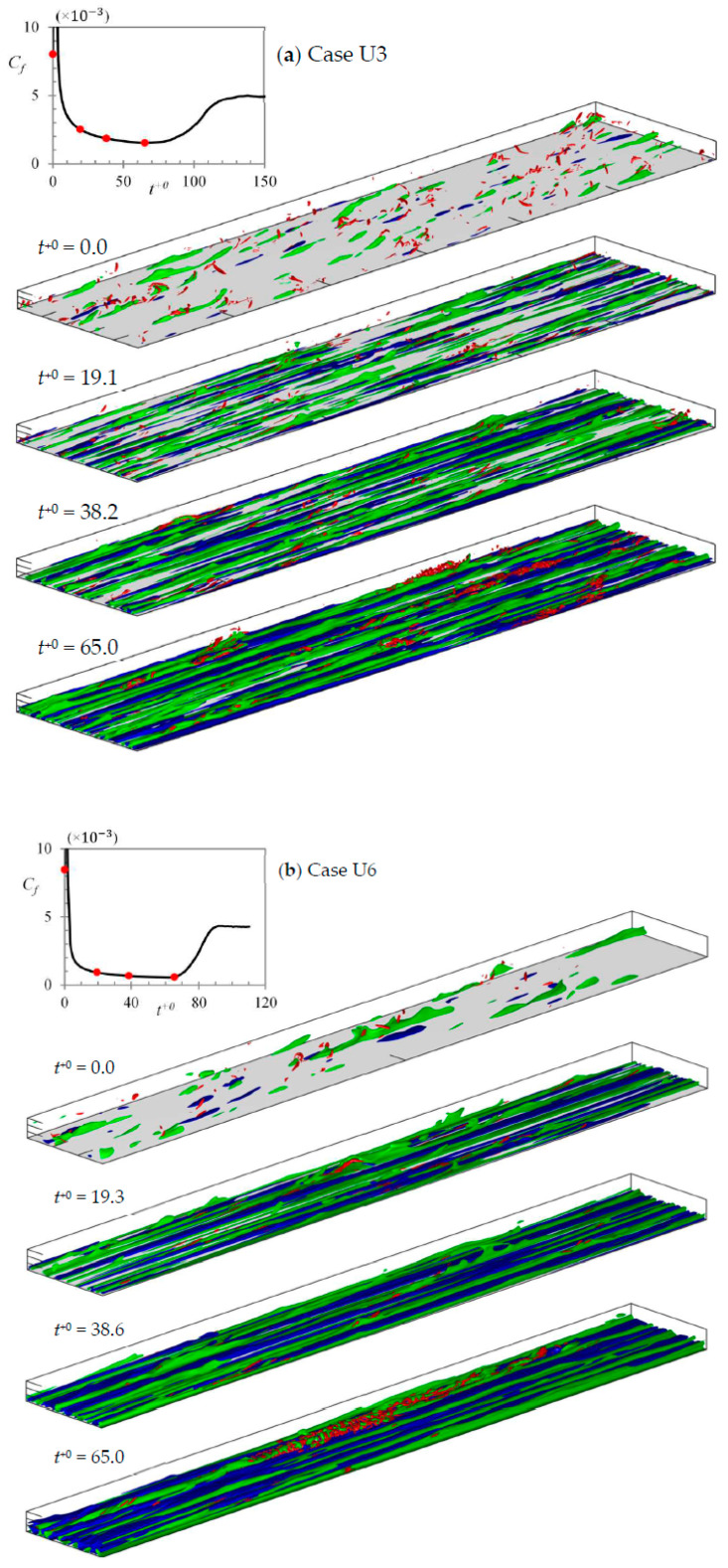
Three dimensional isosurfaces for cases (**a**) U3 and (**b**) U6. Streak structures are shown in blue/green with $u^{\prime }/U_{b0}=\pm 0.35$ and vortical structures are shown in red with $\lambda_{2}/{\left(U_{b0}/\delta \right)}^{2}=-5$. The inset plot shows the development of friction coefficient, with symbols indicating the time instants at which instantaneous plots are presented.

The flow structures at several time instants during the transient period for cases U3 and U6 are presented in Figure 3, using the isosurface plots of $u^{\prime }/U_{b0}$ and $\lambda_{2}/{\left(U_{b0}/\delta \right)}^{2}$. Here, the blue and green isosurfaces are the positive and negative streamwise velocity fluctuations, $u^{\prime }$($=u-\bar{u}$); and red iso-surfaces are vortical structures represented by $\lambda_{2}$, where $\lambda_{2}$ is the second largest eigenvalue of the symmetric tensor $S^{2}+\Omega^{2}$, S and Ω are the symmetric and anti-symmetric velocity gradient tensor ∇u. Figure 3a shows instantaneous plots in the entire domain size (24δ×5δ in X–Z direction) for case U3. However, due to space constraints, only one-third of the domain length (24δ×3δ in X–Z directions) is presented for case U6 in Figure 3b. Also presented in the inset is the development of the friction coefficient for the corresponding wall for a single realization. The symbols indicate the time instants for which the instantaneous plots are shown. The critical times of onset and completion of transition are clearly identifiable from the development of the friction coefficient (He and Seddighi [15]). The time of minimum friction coefficient approximately corresponds to the appearance of first turbulent spots and, hence, the onset of transition; while the time of first peak corresponds to a complete coverage of wall with newly generated turbulence and, hence, the completion time.

It is seen that the response of the transient flow is essentially the same as that described in He and Seddighi [15,16]—a three stage response resembling the bypass transition of boundary layer flows. In the initial flow (at $t^{+0}=0$), patches of high- and low-speed fluctuating velocities and vortical structures are seen, representative of a typical turbulent flow. In the early period of the transient (at $t^{+0}=20$), elongated streaks are formed, represented by alternating tubular structures of isosurfaces of positive and negative $u^{\prime }/U_{b0}$. These structures are similar to those found in the pre-transition regions of the boundary layer flow (Jacobs and Durbin [27]; Matsubara and Alfredsson [28]). The number of vortical structures is also seen to reduce during this stage. Further at $t^{+0}=40$, it seen that the streak structures are further stretched and become stronger. It is noted that in the higher Reynolds number-ratio case, the streaks appear stronger and longer; and the vortical structures appear to reduce by a greater extent—a trend also reported in HS15. New vortical structures start to appear at $t^{+0}=65$, representing burst of turbulent spots which trigger the onset of transition. Afterwards, these turbulent spots grow with time to occupy more wall surface and eventually cover the entire domain signifying the completion of transition. It is again observed that the number of the initial turbulent spots seem to be more scarce for case U6 and some of the streaks extend nearly the entire domain length. Thus, the present domain lengths are sufficiently increased to reduce any effect of the domain size in the higher Reynolds-number ratio cases. This is further demonstrated later in the next section.

**Figure 4:**
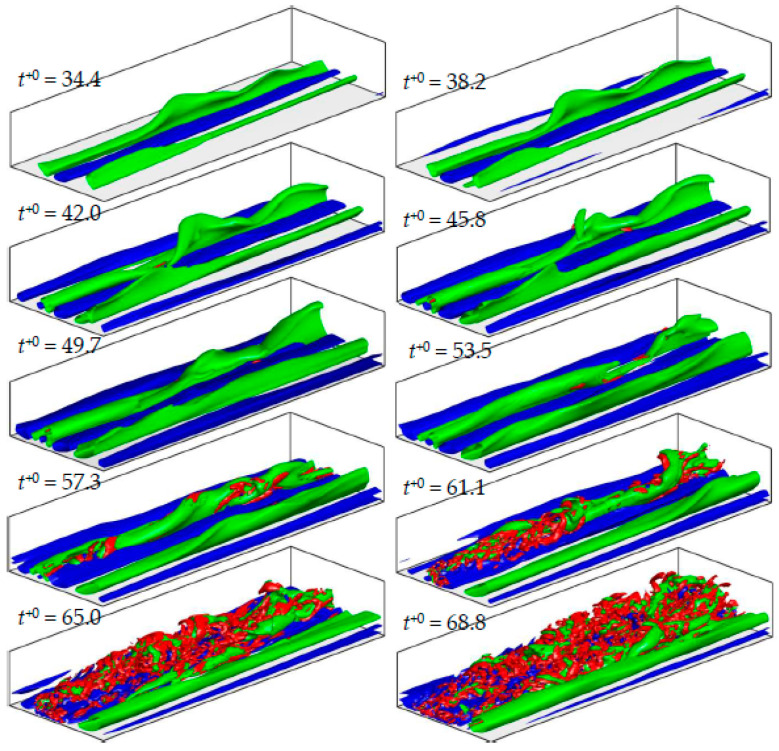
Visualization of streak instability and breakdown in case U3 using a sliding window. 3D iso-surface streak structures are shown in blue/green with $u^{\prime }/U_{b0}=\pm 0.65$, and vortical structures are shown in red with $\lambda_{2}/{\left(U_{b0}/\delta \right)}^{2}=-80$.

In order to visualise the instability and breakdown occurring in the low-speed streak, the site of the initial turbulent spot for case U3 is traced back in time; and a *sliding window* (of size 3δ×1δ in the X-Z direction) is used to follow the event in the domain during the late pre-transition and early transitional period, moving roughly a distance of 1δ downstream per two initial wall-units of time ($\Delta L_{x}/\Delta t^{+0}\sim \;0.5\delta$). Visualisations of 3D isosurface structures inside this window are presented in Figure 4 at several time instants during this period. It is seen that for the most part of the pre-transition period (up to $t^{+0}=49.7$) the streaks undergo elongation and enhancement. At about halfway during pre-transition period, the low-speed streak begins to develop an instability, similar to the sinuous instability of boundary-layer transitional flows (Brandt et al. [29,30,31]; Schlatter et al. [32]). This type of instability is reported to be driven by the spanwise inflections of the streamwise velocity and is characterised by antisymmetric spanwise oscillations of the low-speed streak (Swearingen and Blackwelder [33]). In the late pre-transitional period (about $t^{+0}=57.3$), the streak appears to break down accompanying the generation of some vortical structures. Afterwards, bursts of turbulent structures appear surrounding the low-speed streak site, which continue to grow in size and soon outgrow the size of the window.

**Figure 5:**
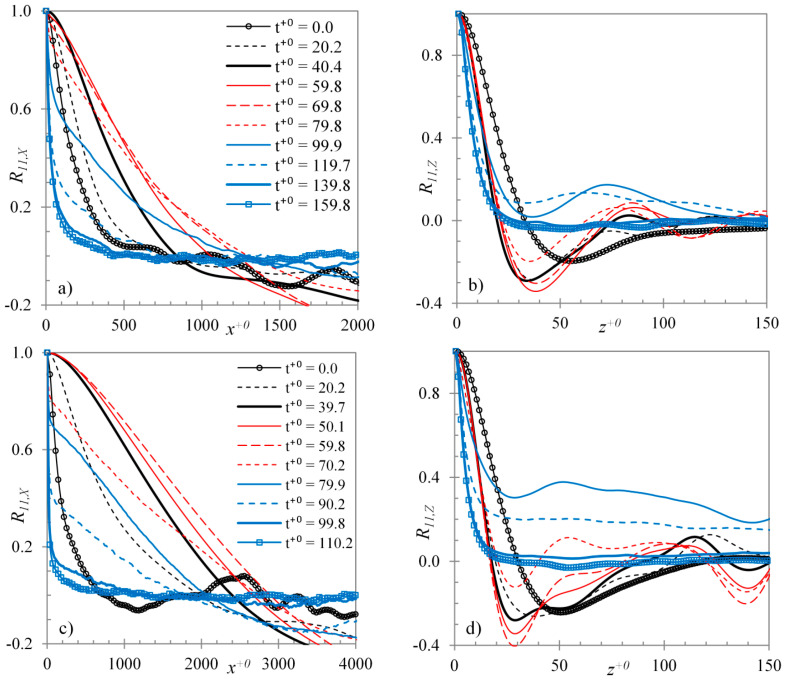
Streamwise velocity autocorrelations at several time instants during the transient for case U3 (**a**,**b**) and U6 (**c**,**d**) in the streamwise (**a**,**c**) and spanwise directions (**b**,**d**) at $y^{+0}=10$.

Overall, it is seen that the features of the transition process become more striking in case U6 than that in U3. The quantitative information about streaks can be obtained by the correlations of the streamwise velocity ($R_{11}$). Correlations in the streamwise direction provide a measure of the length of the streaks, whereas those in the spanwise direction measure the strength and the spacing between streaks. Figure 5 presents these correlations for case U3 (a,b) and U6 (c,d) in the streamwise (a,c) and spanwise directions (b,d). It can be seen from the initial flows (at $t^{+0}=0$) of both cases that the length of the streaks (given by the streamwise correlations) is about 800 wall units (based on the initial flow) and the location of minimum spanwise correlations is about 50 wall units, implying that the spacing of streaks is about 100 wall units. This is representative of a typical turbulent flow. After the start of the transient, these streaks are stretched in the streamwise direction. It is seen that until the end of the pre-transitional period (at $t^{+0}=70-80$), the streaks are stretched to a maximum of 1200 wall units in case U3, whereas to 3000 wall units in case U6. During this time, the spacing between the streaks is reduced to about 75 wall units in case U3, and to 56 wall units in case U6. The minimum value of the spanwise correlations provides a measure of strength of the streaks. It is clearly seen that this value is lower for case U6 in comparison to that in U3. Thus, the streaks in the pre-transitional stage of case U6 are much longer, stronger and more densely packed than those in case U3.

**Figure 6:**
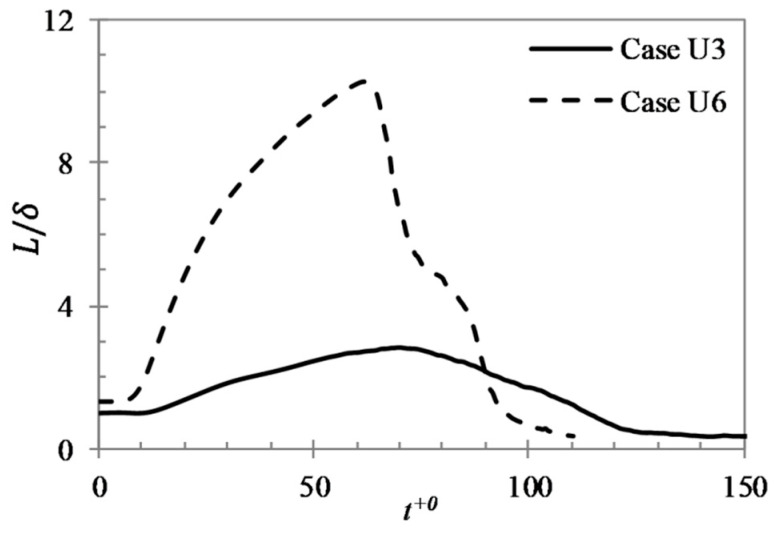
Development of the integral length scale of the flow in U3 and U6.

To further illustrate the development of the flow structures during pre-transition period, the variations of the integral length scales ($L=\int_{0}^{x_{0}}R_{11}dX,$ where $x_{0}$ is the location when $R_{11}$ first reaches zero) in U3 and U6 are shown in Figure 6. It can be seen that the integral length scale increases significantly during the pre-transition period, reaching a peak at the time around the onset of transition. The peak value is over doubled that of its initial value in U3 but around 8 times in U6. This trend is clearly consistent with the streaks observed in Figure 3 and the correlations shown in Figure 5.

**Figure 7:**
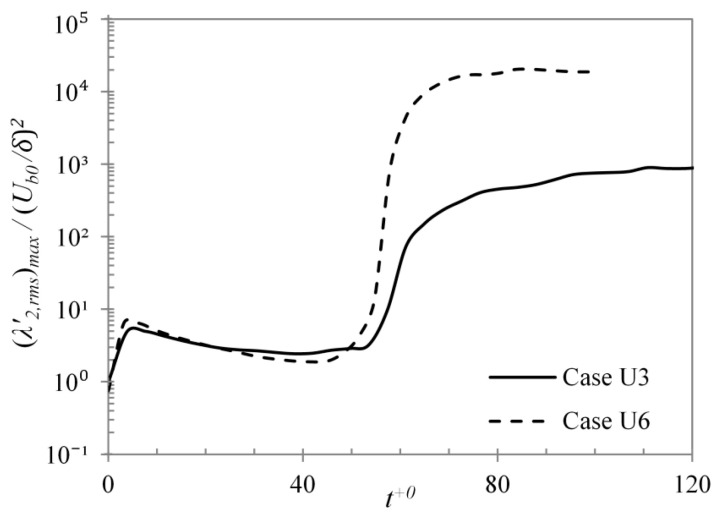
Time development of ${\left(\lambda_{2,rms}^{\prime }\right)}_{max}/{\left(U_{b0}/\delta \right)}^{2}$ during the transient for cases U3 and U6.

The near wall vortical structures were visualised by the $\lambda_{2}$-criterion in Figure 3 and Figure 4 earlier. The same criterion can also be used to get some quantitative information about these structures. Jeong and Hussain [34] noted that $\lambda_{2}$ is positive everywhere outside a vortex core and can assume values comparable to the magnitudes of the negative $\lambda_{2}$ values inside the vortices. Jeong et al. [35] showed that due to significant cancellation of negative and positive regions of $\lambda_{2}$ in the buffer region, a spatial mean $\langle \lambda_{2}\rangle$ was an ineffective indicator of the vortical events. It was reported that the r.m.s. fluctuation of $\lambda_{2}$, $\lambda_{2,rms}^{\prime }$, shows a peak value at $y^{+}\sim \;20$, indicating prominence of vortical structures in the buffer region. Hence, the maximum value of $\lambda_{2,rms}^{\prime }$ can be used to compare the relative strength of these structures in the flow. Figure 7 shows the variation of ${\left(\lambda_{2,rms}^{\prime }\right)}_{max}$ during the transient for the cases U3 and U6. Here, ${\left(\lambda_{2,rms}^{\prime }\right)}_{max}$ is normalised by $U_{b0}/\delta$. It can be seen that in the early period of the transient, the value of ${\left(\lambda_{2,rms}^{\prime }\right)}_{max}$ increases abruptly during the excursion of the flow acceleration (till $t^{+0}\sim \;3$). This is attributed to the straining of near-wall velocity due to the imposed flow acceleration, resulting in distortion of the pre-existing vortical structures and, hence, high fluctuations of $\lambda_{2}$. After the end of the acceleration, the values are seen to gradually reduce, which signify a breakdown of the equilibrium between the near-wall turbulent structures and the mean flow. The formation of high shear boundary layer due to the imposed acceleration causes the high-frequency disturbances to damp and shelters the small structures from the free-stream turbulence. This phenomenon of disruption of the near-wall turbulence is referred to as *shear sheltering* [36]. Later in the late pre-transition stage, ${\left(\lambda_{2,rms}^{\prime }\right)}_{max}$ begins to increase gradually as the new structures begin to form. At the onset of transition, this value increases rapidly due to burst of turbulent spots and generation of new turbulent structures in the flow. The rate of increase of ${\left(\lambda_{2,rms}^{\prime }\right)}_{max}$ can be used to indicate the strength of turbulence generation. It is clearly seen that the rate is higher for case U6, implying a stronger rate of turbulence generation in comparison to case U3.

This trend is similar to that observed in HS15. Therein, the highest Reynolds number ratio case showed a distinct and clear transition process, but the transition of in the lowest ratio case was indiscernible from the instantaneous visualisations. Here, it is seen that as the Reynolds number ratio is increased further (larger than those in HS15), the features of the transition appear to be more striking and prominent. The streaks in the pre-transitional stage are longer and stronger, and are more densely packed, and after the onset of transition the generation of turbulence is stronger.

### 3.2. Correlations of Transition

The onset of transition can be clearly identified using the minimum friction factor during the transient [15]. Thus, a critical time of onset of transition ($t_{cr}$) can be obtained and used to calculate an equivalent critical Reynolds number, $Re_{t,cr}=t_{cr}U_{b1}^{2}/\nu$, where $U_{b1}$ is the bulk velocity of the final flow. Here, the equivalent Reynolds number ($Re_{t}$) can be considered analogous to the Reynolds number $(Re_{x}=xU_{\infty }/\nu$, where is x the distance from the leading edge and $U_{\infty }$ is the free stream velocity) used in the boundary layer flows. It was demonstrated by HS15 that although these two Reynolds numbers cannot be quantitatively compared, $Re_{t}$ has the same significance in the channel flow transition as $Re_{x}$ has in boundary layer transition.

Similar to that in boundary layer transition, the critical Reynolds number here is closely dependent on the initial ‘free-stream turbulence’ and can be represented by:

(7) $$Re_{t,cr}=1340\;Tu_{0}^{-1.71}$$

**Figure 8:**
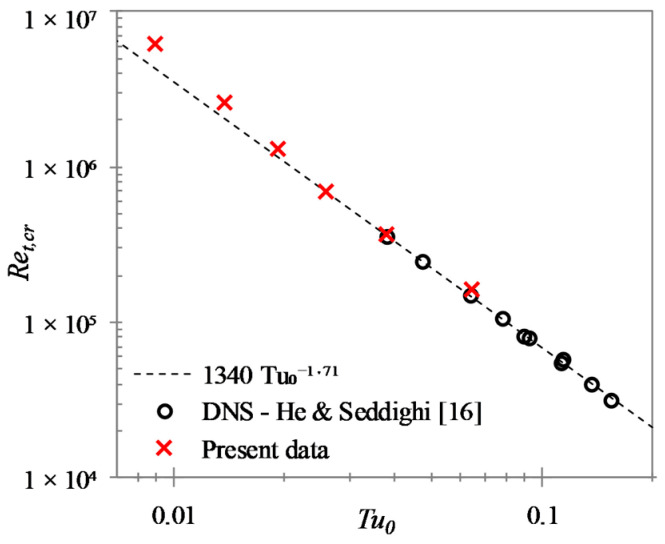
Dependence of equivalent critical Reynolds number on initial turbulence intensity.

Figure 8 shows the relation between the equivalent critical Reynolds number and the initial turbulence intensity for the present LES cases and the DNS cases of HS15 for comparison. The present data follows the Equation (7) established from the higher turbulence intensity cases (U1–U4). However, the lower turbulent intensity cases, namely cases U5 and U6, are seen to diverge from this relation, with transition occurring at higher $Re_{t}$ values.

**Figure 9:**
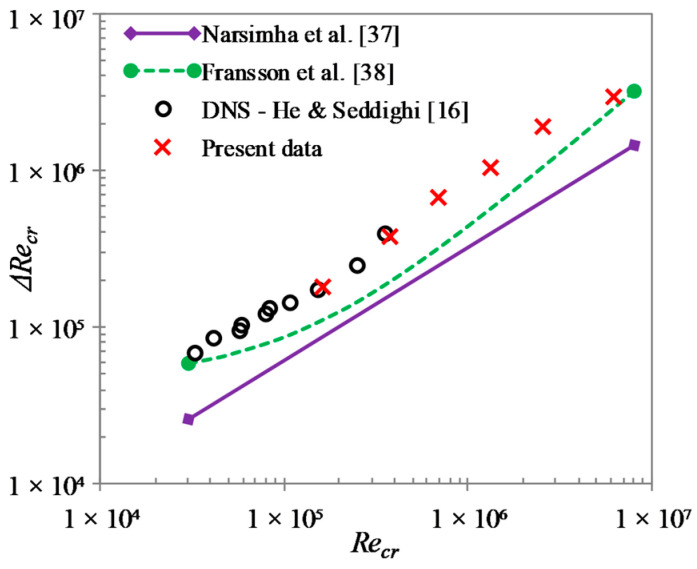
Relationship between transition period Reynolds number and critical Reynolds number.

Similar to onset of transition, friction factor can also be used to determine the time of completion of the transition process ($t_{turb}$). By assuming that the transition is complete when the friction factor reaches its first peak, a transition period can thus be obtained ($\Delta t_{cr}=t_{turb}-t_{cr}$). The relation between the equivalent transition period Reynolds number ($\Delta Re_{t,cr}=\Delta t_{cr}U_{b1}^{2}/\nu$) and the critical Reynolds number is presented in Figure 9. Also shown in the figure is the power-relation for transition length of boundary layer flows by Narasimha et al. [37], and the linear-relation between the same by Fransson et al. [38]. It should be noted that $Re_{cr}$ in the figure denotes $Re_{t,cr}$ and $Re_{x,cr}$ for the boundary layer flow and the transient channel flow, respectively. It is seen that, similar to the findings of HS15, the presented data is reasonably well predicted by the boundary layer correlations if a factor of 0.5 is applied to the present $\Delta Re_{t,cr}$. However, the present data seem to suggest a power-relation between $\Delta Re_{t,cr}$ and $Re_{t,cr}$, similar to that of Narasimha et al. [37].

**Figure 10:**
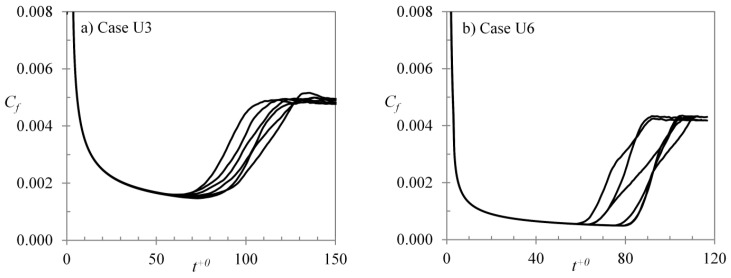
Deviations in different realizations for cases (**a**) U3; and (**b**) U6.

**Figure 11:**
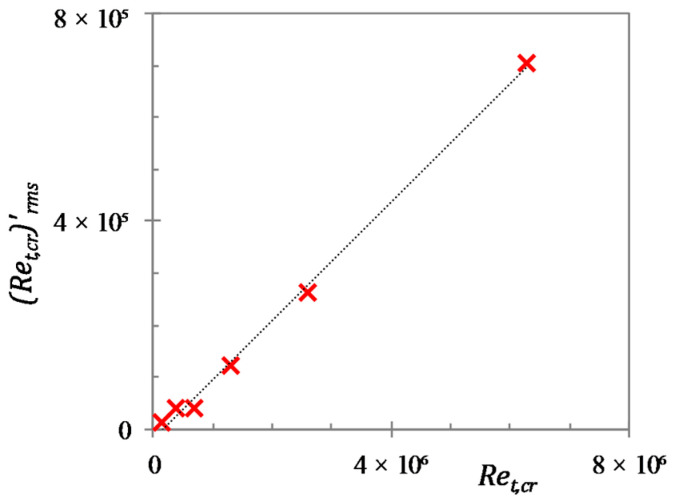
Deviations observed in the equivalent critical Reynolds number for the present cases.

The critical Reynolds number discussed above is naturally a statistical concept. In each flow realisation, the generation of turbulence spots and transition to turbulence may vary significantly around the ”mean” $Re_{t,cr}$. The generation of turbulent spots is to some extent dependent on the initial flow structures. Due to this, the time and spatial position at which the generation of turbulent spot occurs can vary with different initial flow fields. Thus, several simulations have been run for each case, each starting from a different initial flow field to arrive at an average critical and transition period Reynolds numbers. It is observed that there are large deviations in the critical Reynolds number for different realizations, and for the top and bottom walls of a single realization for the present cases. Friction factor histories for both walls of different realizations for cases U3 and U6 are presented in Figure 10. It is seen that the deviations in the critical time are larger in case U6 than those in case U3. The degree of the scatters of the critical Reynolds number for the present cases is found to be linearly proportional to the average value. As shown in Figure 11, the r.m.s. of fluctuation of the critical Reynolds numbers are roughly 10% of the average value.

**Figure 12:**
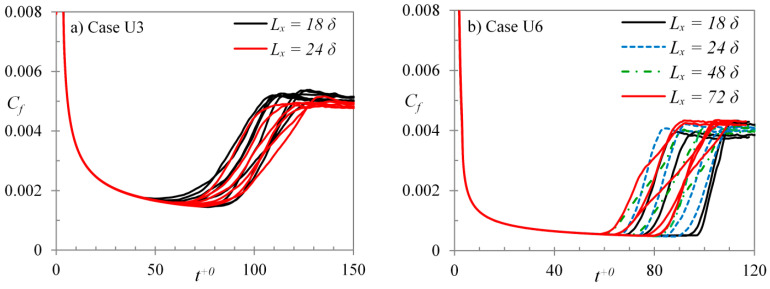
Friction factor developments using different domain lengths for cases (**a**) U3; and (**b**) U6.

**Figure 13:**
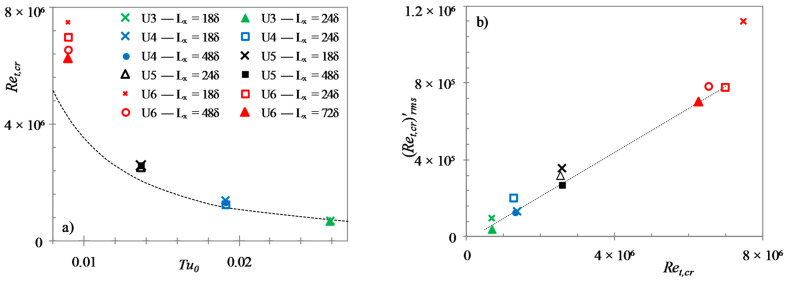
Effect of domain length on (**a**) the critical Reynolds number; and (**b**) r.m.s. fluctuation of critical Reynolds number. Here, the largest domain length in each case is marked with a solid/filled symbol.

The present higher Reynolds number ratio cases (namely, case U3–U6) were also simulated with different domain lengths to see its effect on the onset of transition and the deviations observed in its predicted critical time. Case U3 was performed with two different domain lengths—18δ and 24δ; cases U4 and U5 each with three lengths—18δ, 24δ and 48δ; whereas, case U6 with four different lengths—18δ, 24δ, 48δ and 72δ. It should be noted that the spatial resolution for different domain lengths of each case was kept roughly the same so that an appropriate comparison can be made. Figure 12 presents the friction factor histories for both walls of every realization for cases U3 and U6. It is observed that as the domain length is increased, the spread of deviations of $Re_{t,cr}$ for multiple realizations is slightly decreased. For case U6, the spread of deviations for the two larger domain lengths is almost identical. Hence, it can be deduced that the effect of domain lengths is very small for the two larger domains. The average critical Reynolds numbers and their r.m.s. deviations, for different domain lengths of cases U3–U6 are presented in Figure 13a,b, respectively. It is clearly seen that the critical Reynolds numbers obtained using different domain lengths for U3 to U5 are largely the same in each case, hence demonstrating the smallest domain size is adequate in capturing the transition time. It is also seen that the larger the domain or the smaller the Reynolds number ratio, the smaller the r.m.s. of $Re_{t,cr}$ suggesting less realisations are needed for such cases to obtained a reliable $Re_{t,cr}$. For case U6, the critical Reynolds number observed decreases slightly as the domain length is increased even for the largest domain sizes (Figure 13a). The streaks are very long and the initial turbulence spots generated are spares in a high *Re*-ratio flow, and hence a larger domain is required.

### 3.3. Turbulent Fluctuations

**Figure 14:**
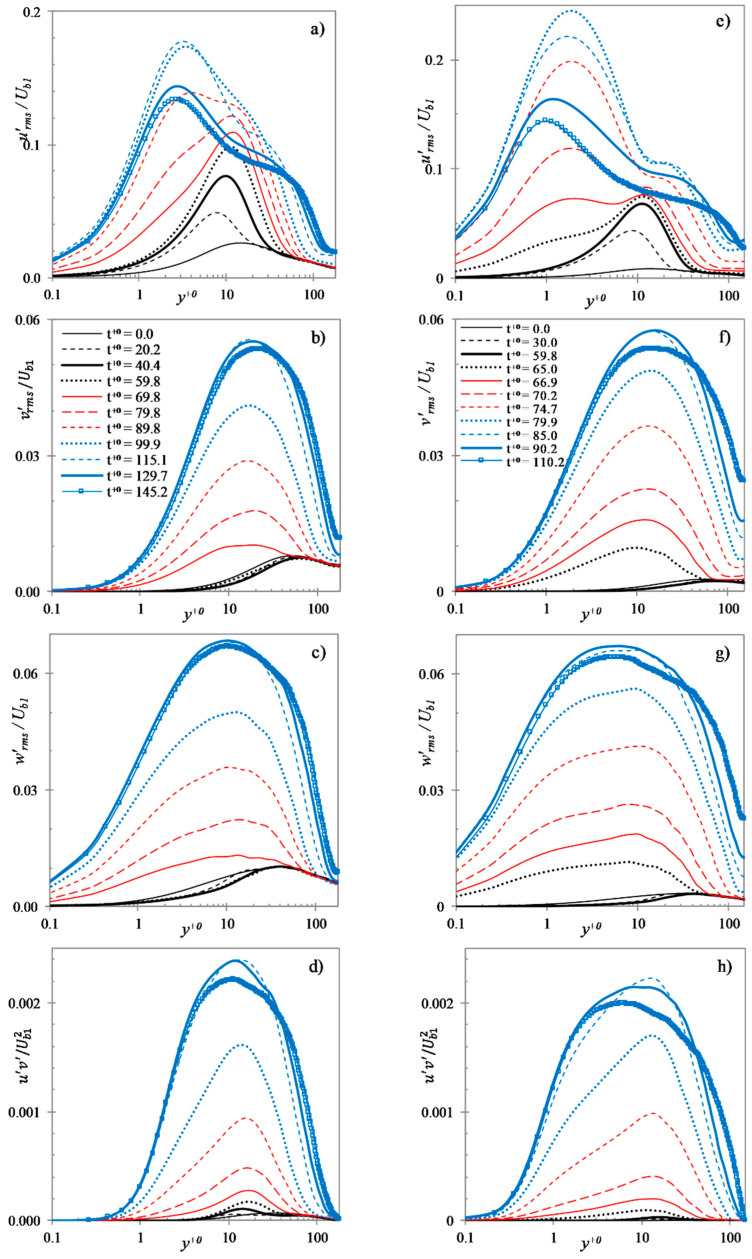
R.M.S. fluctuating velocities and Reynolds stress at several time instants during the transient in cases U3 (**a**–**d**) and U6 (**e**–**h**).

Figure 14 presents the development of r.m.s. fluctuating velocity profiles for cases U3 and U6. As shown earlier in Figure 3, the critical time for both cases is approximately $t^{+0}=65$, while the completion time for U3 and U6 are roughly $t^{+0}=120$ and 85, respectively. It can be seen that following the start of the transient, $u_{rms}^{\prime }$ progressively increases in the wall region and maintains this trend until the onset of transition. On the other hand, the transverse components ($v_{rms}^{\prime }$ and $w_{rms}^{\prime }$) reduce slightly from the initial values and remain largely unchanged until the onset of transition. The Reynolds stress increases very slightly during this period, exhibiting a behaviour that is closer to that of the transverse components than to that of the normal component. During the transition period, $u_{rms}^{\prime }$ further increases rapidly in the near wall region. It is interesting to note that case U6 clearly shows formation of two peaks of $u_{rms}^{\prime }$ during this period ($t^{+0}=67-85$), however, case U3 shows a single peak. Similar double-peaks are also observed in cases U4 and U5 (not shown). The first peak, very close to the wall, is formed rapidly during the transitional period, increasing from very low initial values; whereas, the second peak, farther from the wall, is only slightly higher than that at the point of onset of transition. At the end of the transitional period, $u_{rms}^{\prime }$ reduces and approaches its final steady value. During the transition period the transverse components increase rapidly and monotonically to peak values, showing a slight overshoot towards the end of the transient. The feature of two peaks is not shown by these components.

To further analyse the origin and location of the two peaks in the present cases, the *conditional sampling* technique of Jeong et al. [35] and Talha [39] is used. Here, the r.m.s. fluctuation of $\lambda_{2}$, $\lambda_{2,rms}^{\prime }$, is used to distinguish the ‘active areas’ of turbulent generation from the ‘inactive areas’. It should be noted that this technique is performed to separate the active areas of turbulence generation in the *x-z* domain, rather than in the wall-normal direction. The criterion is based on the comparison of a local r.m.s. fluctuation of $\lambda_{2}$ with a *base* value. The base value chosen here is the $\lambda_{2,rms}^{\prime }$ of the entire *x*–*z* plane at the critical time of onset of transition. Similar to that used by Jeong et al. [35], a window of size ($\Delta x^{+}$, $\Delta z^{+}$) = (120, 50) is used to determine the local r.m.s. fluctuation. The r.m.s. fluctuation is computed in the *x-z* direction and, thus, is a function of *y*. The values are then summed in the wall-normal direction for 50 wall units and compared with each other. The criterion for determining active area reads:

(8) $$\overset{N_{y}}{\underset{j=1}{\sum }}\tilde{\lambda_{2,rms}^{\prime }}\geq 0.1\overset{N_{y}}{\underset{j=1}{\sum }}\lambda_{2,rms,cr}^{\prime }$$

where $\tilde{\lambda_{2,rms}^{\prime }}$ is the local r.m.s. fluctuation value within the window, $\lambda_{2,rms,cr}^{\prime }$ is the r.m.s. fluctuation value of the entire *x*–*z* plane at the onset of transition, and $N_{y}$ is the number of control volumes in the wall region of $y^{+}<50$. It should be noted that the wall units are based on the average friction velocity of all active areas in the domain. Hence, the determination of the window size is an iterative process. Number of iterations was kept such that the change in active area determination for successive iterations was less than 0.1%. It is seen in Figure 7 that the value of ${\left(\lambda_{2,rms}^{\prime }\right)}_{max}$ at the onset of transition ($t^{+0}=65$) reaches close to the fully turbulent value. Thus, the criterion (Equation (8)) distinguishes the areas of *newly* generated turbulence in the transitional period. For any time before the onset of transition or after the completion of transition, the criterion gives 0% or 100% (of *x*–*z* domain), respectively, as active areas of turbulence generation.

**Figure 15:**
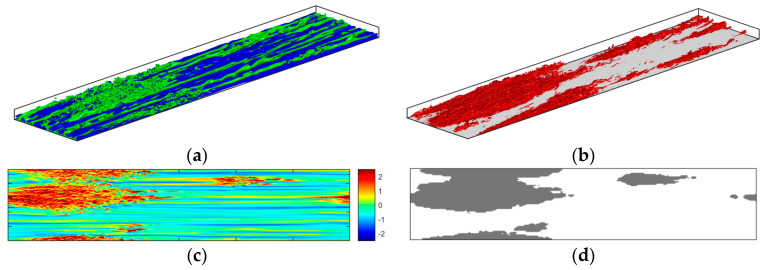
Instantaneous flow for case U3 at $t^{+0}=89.8$ (**a**) isosurface structures of $u^{\prime }/U_{b0}=\pm 0.35$; (**b**) isosurface structures of $\lambda_{2}/{\left(U_{b0}/\delta \right)}^{2}=-5$; (**c**) contours of streamwise fluctuating velocity $u^{\prime }/U_{b0}$ at $y^{+0}=5$; (**d**) active region of turbulence production (shown in gray) determined using Equation (7).

The above scheme is used to distinguish the active areas of turbulent generation for all the present cases. At the beginning of the transient, the entire wall surface is classified as inactive region. At the onset of transition, the active region emerges at the location of the turbulent spot burst. During the transitional period, the active area grows in size and eventually covers the entire wall surface at the end of transitional period. To validate the above criterion, the instantaneous flow for case U3 during transitional period (at $t^{+0}=89.8$) is presented in Figure 15. The instantaneous 3D iso-structures of $u^{\prime }$ and $\lambda_{2}$ are presented in Figure 15a,b, respectively. Figure 15c shows the instantaneous contours of $u^{\prime }$ at $y^{+0}=5$, and Figure 15d shows the approximation of the active wall surface determined using Equation (7). It is clearly seen that the present scheme is suitable to capture the active areas of turbulent production during the transition. Although the edges of active regions may be smeared somewhat, any uncertainties caused to the active/inactive areas are negligible.

**Figure 16:**
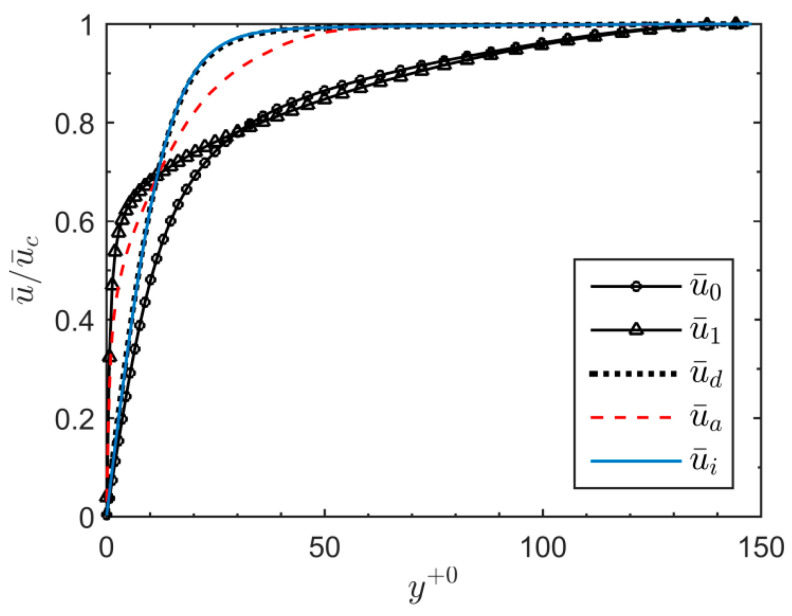
Conditionally-averaged velocity profiles of the active (${\bar{u}}_{a}$) and inactive regions (${\bar{u}}_{i}$), along with the domain-averaged (${\bar{u}}_{d}$) for case U6 at $t^{+0}=67.5$. Also shown are the initial (${\bar{u}}_{0}$) and final (${\bar{u}}_{1}$) steady flow profiles, for comparison.

**Figure 17:**
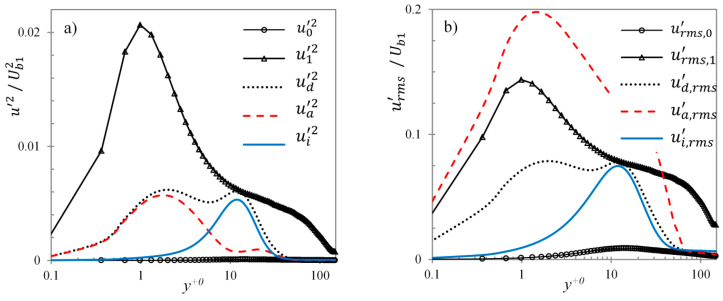
(**a**) Domain-averaged velocity fluctuation energy ($u_{d}^{\prime 2}$), with contributions from the active ($u_{a}^{\prime 2}$) and inactive ($u_{i}^{\prime 2}$) regions for case U6 at $t^{+0}=67.5$, and (**b**) conditionally-averaged velocity fluctuations of the active ($u_{a,rms}^{\prime }$) and inactive regions ($u_{i,rms}^{\prime }$), along with the domain average ($u_{d,rms}^{\prime }$). Also shown in each plot are the domain-averaged initial (subscript 0) and final (subscript 1) steady profiles.

Conditionally-averaged turbulent statistics for the active and inactive areas thus obtained are used to investigate the turbulent intensity contributions from each region. First, the statistics for case U6 at $t^{+0}=67.5$ are presented where the double peak first seems to emerge. At this instant, active region constitutes only 5% of the wall surface. Figure 16 presents the conditionally-averaged velocity profiles, ${\bar{u}}_{a}$ and ${\bar{u}}_{i}$ for the active and inactive regions, respectively, along with the domain-averaged velocity profile, ${\bar{u}}_{d}$. It can be seen that the profiles of the two regions are very different. The inactive region profile resembles that of the pre-transition period, exhibiting a plug-like response to the acceleration, with profile flat in the core. The active region profile, however, has developed farther away from the wall and the near-wall shear resembles that of the final steady flow. The conditionally-averaged streamwise velocity fluctuation profiles at this time are presented in Figure 17. The contributions of fluctuation energy ($\bar{u^{\prime 2}}$) from active/inactive regions to the domain-averaged profile are shown in Figure 17a, whereas, the conditionally-averaged r.m.s. fluctuation profiles ($u_{rms}^{\prime }$) within these regions are shown in Figure 17b. It is clear from Figure 17a that the double peaks in the streamwise fluctuations is the net effect of two separate peaks from two separate regions of the flow, i.e., the active and inactive regions. The near-wall peak originates from the active region whereas that the peak further away from the wall originates from the inactive region. The former (located at $y^{+0}\sim \;1.2$ or $y^{+1}\sim \;15$) is attributed to the burst of new turbulent structures in the active region with its *y-*location consistent with that of the final steady flow, whereas, the latter (located at $y^{+0}\sim \;12$) is the contribution of the elongated streaks in the inactive region. It should be noted that active area profile, $u_{a}^{\prime 2}$, in Figure 17a too has a local second peak further away from the wall (around $y^{+0}\sim \;20$). This is merely a numerical feature due to the method employed in the calculation, where the fluctuation is calculated with respect to the domain-averaged mean profile i.e., $u_{a}^{\prime 2}=\langle {\left(u_{a}-{\bar{u}}_{d}\right)}^{2}\rangle$ and $u_{i}^{\prime 2}=\langle {\left(u_{i}-{\bar{u}}_{d}\right)}^{2}\rangle$, where 〈 〉 denotes a spatial average in the homogeneous (*x*–*z*) plane. This, however, is not an appropriate representation of the conditionally-averaged fluctuation energy because the domain-averaged profile varies from the conditionally-averaged profiles of the active and inactive regions (as seen in Figure 16). To further support this statement, conditionally-averaged r.m.s. fluctuation profiles within these two regions are presented separately in Figure 17b. Here, the velocity fluctuation is calculated with respect to the conditionally-averaged mean flow, i.e., $u_{a,rms}^{\prime }={\left(u_{a}-{\bar{u}}_{a}\right)}_{rms}$ and $u_{i,rms}^{\prime }={\left(u_{i}-{\bar{u}}_{i}\right)}_{rms}$. It is clear that the active region profile, here, shows a single peak consistent with the final steady profile.

**Figure 18:**
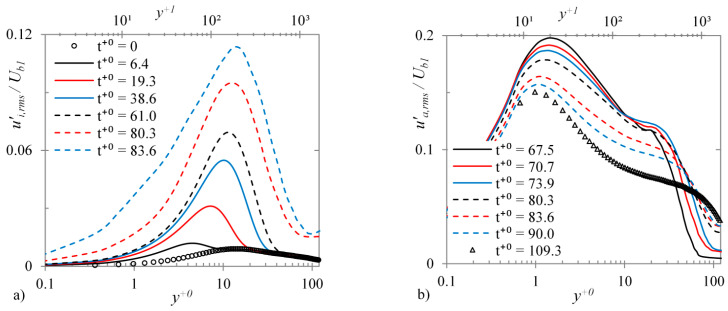
R.M.S. streamwise fluctuating velocity profiles at several time instants during the transient for (**a**) inactive and (**b**) active regions for case U6.

Now, the development of these conditionally-averaged r.m.s. fluctuation profiles during the transient is presented in Figure 18. As shown earlier in Figure 3, the critical times of onset and completion of transition for case U6 are roughly $t^{+0}=65$ and 85, respectively. It is seen that the inactive region profiles increase monotonously from the beginning of the transient until the end of the transitional period. The peak of the profile originates at $y^{+0}\sim \;5$ and moves further away from the wall during the transient, reaching $y^{+0}\sim \;12$ until the end of the transitional period. On the other hand, the active region profile is generated at the point of onset of transition which thereafter reduced gradually during the transitional period. The peak of this profile originates at $y^{+0}\sim \;1.3$ ($y^{+1}\sim \;20$) at the onset of transition and only moves slightly towards the wall during the transitional period and the post-transition period until it settles to the final steady value at $y^{+0}\sim \;1$ ($y^{+1}\sim \;14$).

**Figure 19:**
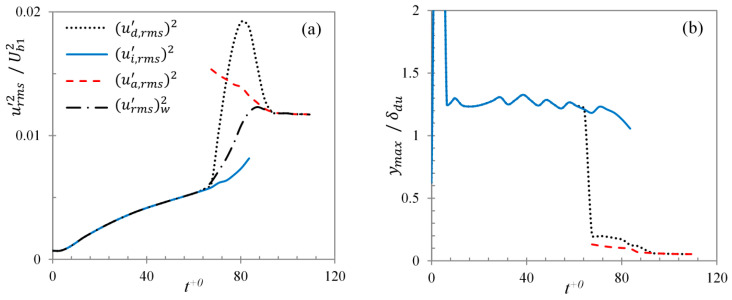
Conditionally-averaged (**a**) maximum energy growth and (**b**) the *y*-location of its peak, for case U6.

The maximum streamwise energy growth, $u_{rms,max}^{\prime 2}\left(=max_{y}{\left\{u_{rms}^{\prime }\right\}}^{2}\right)$, and the *y-*location of its peak for the two different regions of case U6 is presented in Figure 19a,b, respectively. The domain-averaged energy, ${\left(u_{d,rms}^{\prime }\right)}^{2}$, similar to that in DNS cases of HS15, exhibits an initial delay following the start of the transient which is attributed to an early receptivity stage [38]. During the pre-transitional period, the energy increases linearly with time until the onset of transition. At this point, the energy increases rapidly owing to the burst of ‘new’ turbulence, overshooting the final steady value and reaching a peak around the end of the transitional period and thereafter reducing to reach the final steady value. It is seen that the energy growth in the inactive region, ${\left(u_{i,rms}^{\prime }\right)}^{2}$, grows linearly even after the onset of transition and continues to do so until the end of the transitional period. This is expected as the burst of turbulence generation occurs only in the active region, while the inactive region is dominated by the stable streaky structures which continue to develop further. Energy in the active region ${\left(u_{a,rms}^{\prime }\right)}^{2}$, on the other hand, is generated at the onset of transition at a value much higher than the final steady value which gradually reduces until the end of the transitional period and reaches the final steady value. It is worth noting that the sharp increase and the high peak observed in the maximum domain-averaged energy during the transitional period is only a numerical feature arising due to the method of statistical calculation. The domain-averaged energy comprises of the turbulent fluctuations from both the active and inactive regions calculated with respect to the domain-averaged mean velocity, resulting in high values of fluctuations. A more suitable representation during the transitional period is a weighted-average of the fluctuation energy, ${\left(u_{rms}^{\prime }\right)}_{w}^{2}=\alpha \cdot {\left(u_{a,rms}^{\prime }\right)}^{2}+\left(1-\alpha \right)\cdot {\left(u_{i,rms}^{\prime }\right)}^{2}$, where subscript ‘w’ denotes the *weighted-average*, and α is the *active* fraction of wall surface (plotted in Figure 19a). It is clear that the average energy of the streamwise fluctuations show only a slight overshoot during the transitional period. The overshoot is attributed to the increasingly dominant effect of the active region during this period, while the slight decrease towards the end of the transitional period is attributed to the redistribution of streamwise energy to transverse components.

The *y*-location of the peak of streamwise energy, normalised by the displacement thickness of the velocity field ($\delta_{u}$), are shown in Figure 19b. It should be noted that conditionally-averaged peak energy location is normalised by $\delta_{u}$ of respective conditionally-averaged profile. Immediately after the commencement of the transient, a sharp increase is seen in $y/\delta_{u}$ value of the peak location in the inactive region. This is attributed to the formation of a new thin boundary layer of high shear due to the imposed acceleration, and hence a smaller boundary layer thickness. Further in the pre-transition period the peak of the energy profile is seen to scale with the displacement thickness, rather than the inner scaling, which is atypical of turbulent flows. The location of the peak maintains at ~1.25$\delta_{u}$ up until the onset of transition, implying that the streamwise energy grows with the growth of the time-developing boundary layer—a feature observed in bypass transitional flow. The peak in the inactive region is seen to largely maintain its location after the onset of transition showing only a slight decrease towards the end of the transitional period. The peak in the active region appears very close to the wall, typical of high Reynolds number turbulent flows. The displacement thickness of turbulent boundary layer in the active region increases with time as it becomes fully developed. Thus, the peak of the streamwise energy appears to move from ~0.12$\delta_{u}$ at the point of onset of transition to ~0.06$\delta_{u}$ at the end of the transient. During the pre-transitional period, the entire wall surface is inactive region, thus the domain-averaged peak follows the same trend as that in the inactive region. At the onset of transition, the active region peak, which appears much closer to the wall, has a much higher value than that in inactive region. At this point, the domain-averaged peak is dominated by the active region energy, and seems to follow the location of the active region peak. From the point of onset of transition until the end of transitional region, both active and inactive regions co-exist and exhibit separate developments of their respective streamwise energies. At the onset of transition, there is a large difference between the peak energy of the active region and that in the inactive region. Thus, even though the active region covers only a small fraction of the wall surface, the domain-averaged energy shows a dominant contribution from active region in the near-wall region. The difference between wall normal locations of the peak energies for the two regions also plays a role in enhancing the difference between two separate contributions. The domain-averaged profile, thus, shows the net effect of two peaks. The peak closer to the wall is attributed to the turbulent spots generated at the onset of transition, whereas, the one further away from the wall is attributed to the elongated streaks. In the late transitional period, most of the wall surface is covered with the new turbulence, thus reducing the area of the inactive region. This results in a decreasing contribution of the inactive region, until the inactive region energy is completely masked by the active region energy. At the end of the transitional period, the entire wall becomes the active region with only a single peak in the entire domain. Thus, from the late-transitional period until the end of the transient, the domain-averaged profile shows only a single peak (i.e., peak associated with the generation of ‘new’ turbulence in the active region). Separate developments of active and inactive regions exist in all the present cases (U1–U6). However, the feature of double-peaks is clearly visible only in cases U4–U6.

**Figure 20:**
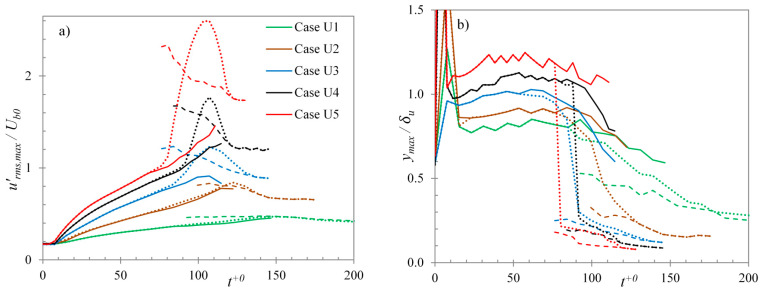
Domain- and conditionally-averaged (**a**) maximum streamwise fluctuations; and (**b**) the *y*-locations of their peaks, for cases U1–U5 (Dotted: domain-averaged; solid: inactive region; dashed: active region).

Figure 20a,b show the maximum streamwise fluctuations and the *y*-location of the peaks for the cases U1–U5, respectively. Here, the dotted lines represent the domain-averaged values, and the solid and dashed lines represent the conditionally-averaged inactive and active region values, respectively. It can be seen that at the onset of transition (time at which active region value appears), the difference between the maximum fluctuations of the active and inactive regions is very small for cases U1–U3. The resulting active region contribution to the domain-averaged value in the near-wall region is also less than that of the inactive region. Thus, the net effect in the domain-averaged value for these cases shows only a single peak during the transitional period—the peak corresponding to the inactive region; while the active region peak is masked by the inactive region fluctuations. Later in the transitional period, when the active region grows in size, its contribution becomes comparable to that of the inactive region. However, due to close proximity of the two peaks, the domain-averaged profile appears as a single peak. Again, in the late transitional period, the area occupied by the inactive region becomes increasingly small and its contribution to the calculation of turbulent quantities diminishes. The area is then dominated by ‘new’ turbulence in the active region. Thus, these cases show a single peak in the streamwise fluctuation during the entire transient period.

The two peaks shown by the streamwise component during the transient of high Re-ratio cases are very similar to the experimental results of Greenblatt and Moss [9]. However, in their case the peaks farther from the wall were formed at $y^{+0}=300$, which persisted until the end of the unsteady flow period. Due to limitations in their near-wall velocity data, the full magnitude and location of the near-wall peak was not captured. Although the present results do show two peaks, a direct comparison of these with the two peaks of Greenblatt and Moss [9] might not be appropriate due to the large differences in the initial and final Reynolds numbers. It is possible that their peak farther from the wall (at $y^{+0}=300$) is a high Reynolds number effect.

## 4. Conclusions

LES has been performed for step-like accelerating channel flow with a Reynolds number ratio up to ~19 (or $Tu_{0}$ of 0.9%). Similar to the findings of HS15, the present cases with higher Reynolds number ratio also show a three-stage response resembling that of the bypass transition in boundary layer flows. However, the features of transition become more striking when the Reynolds number ratio increases—the elongated streaks in the pre-transitional period become increasingly longer and stronger, and the turbulent spots generated at the initial stage at the onset of transition become increasingly sparse. For the lower turbulence intensity cases, the critical Reynolds number of transition is seen to diverge from the DNS trend of HS15. It was observed that there are large deviations of the critical Reynolds number for different realizations of each case. For the present cases, these deviations increase linearly with the mean value. It is noted that the length of the domain needs to be sufficiently large to accurately capture the transition time when the Reynolds number ratio is high. The present cases are performed using different domain lengths to verify the adequacy of the domain lengths.

The higher Reynolds number ratio cases are found to show double peaks in the transient response of streamwise fluctuations profiles shortly after the onset of transition. A conditional sampling technique is used to further investigate the streamwise fluctuations in all the cases. The wall surface is classified into active and inactive regions of turbulence generation based on a $\lambda_{2}$-criterion. Conditionally-averaged turbulent statistics, thus obtained, are used to show that the fluctuation energies in the two regions undergo separate developments during the transitional period. For the high-Reynolds number ratio cases, the two peaks in the domain-averaged fluctuation profiles originate from the separate contributions of the active and inactive regions. The peak close to the wall is attributed to the generation of ‘new’ turbulence in the active region; whereas the peak further away from the wall is attributed to the elongated streaks in the inactive region. In the low-Reynolds number ratio cases, the peaks of the two regions are masked by each other during the entire transient, resulting in a single peak in the domain-averaged profile.

## Figures and Tables

**Table 1:** Present accelerating flow cases with the DNS cases of He & Seddighi (2013, 2015) for comparison.

Case	$Re_{0}$	$Re_{1}$	$\frac{Re_{1}}{Re_{0}}$	$Tu_{0}$	Grid	$L_{x}/\delta$	$L_{z}/\delta$	$\Delta x^{+1}$	$\Delta z^{+1}$	$\Delta y_{c}^{+1}$
HS13 [15]	2825	7404	2.6	0.065	512 × 200 × 200	12.8	3.5	11	7	7
HS15 [16]	2800	12,600	4.5	0.038	1024 × 240 × 480	18	5	12	7	10
U1	2825	7400	2.6	0.065	192 × 128 × 160	12.8	3.5	28	9	13
U2	2825	12,600	4.5	0.038	450 × 200 × 300	18	5	26	11	13
U3	2825	18,500	6.5	0.026	1200 × 360 × 540	24	5	19	9	10
U4	2825	25,000	8.8	0.019	2400 × 360 × 360	48	3	24	10	13
U5	2825	35,000	12.4	0.014	2400 × 360 × 360	48	3	32	13	18
U6	2333	45,000	19.3	0.009	2400 × 360 × 360	72	3	60	17	22
